# Association of the Fibrosis-4 Index With Indices of Atherosclerosis in Patients With Type 2 Diabetes Mellitus

**DOI:** 10.7759/cureus.117699

**Published:** 2026-10-09

**Authors:** Ahmad Farooq, Mohammed Mohsin Raza, Nawaal Saeed, Shehriyar Khan, Muhammad Shahid Nawaz Khan

**Affiliations:** 1 Internal Medicine, Cwm Taf Morgannwg University Health Board, Abercynon, GBR; 2 Medicine, Sheikh Zayed Medical College and Hospital, Rahim Yar Khan, PAK; 3 Internal Medicine, Liver Clinic, Lahore, PAK; 4 Internal Medicine, Saidu Teaching Hospital, Swat, PAK; 5 Geriatric and General Internal Medicine, Cambridge University Hospitals NHS Foundation Trust, Cambridge, GBR; 6 Medicine, Tertiary Care Hospital Nishtar-II, Multan, PAK

**Keywords:** arterial stiffness, atherosclerosis, bapwv, carotid intima-media thickness, carotid plaque, fib-4, type 2 diabetes mellitus

## Abstract

Background

In patients with type 2 diabetes mellitus (T2DM), the Fibrosis-4 (FIB-4) index can be a simple indicator of vascular risk. This study aimed to assess the association between the FIB-4 index and subclinical vascular disease in patients with T2DM using mean carotid intima-media thickness (CIMT) as the primary outcome and maximum CIMT, carotid plaque, and brachial-ankle pulse wave velocity (baPWV) as secondary vascular outcomes.

Methodology

This analytical cross-sectional study was conducted in a hospital setting at Sheikh Zayed Medical College and Hospital, Pakistan, between April 2024 and March 2026. A total of 250 individuals with T2DM were consecutively recruited. FIB-4 was computed using age, aspartate aminotransferase, alanine aminotransferase, and platelet count. Maximum CIMT, carotid plaque, and baPWV were secondary outcomes to mean CIMT. Both a multivariable regression analysis and a correlation analysis were performed.

Results

The mean age of the 250 participants was 58.7 ± 10.4 years, and 139 (55.6%) were men. The FIB-4 index was 1.71 ± 0.92 on average. Carotid plaque was present in 71 (28.4%) participants, and the mean CIMT, maximum CIMT, and baPWV were 0.78 ± 0.15 mm, 1.02 ± 0.31 mm, and 1,742 ± 348 cm/s, respectively. Across the low, intermediate, and high FIB-4 groups, mean CIMT increased considerably (0.72 ± 0.11, 0.81 ± 0.14, and 0.91 ± 0.17 mm, respectively; p < 0.001). Carotid plaque was present in 20 (17.9%), 41 (34.5%), and 10 (52.6%) participants in the low, intermediate, and high FIB-4 groups, respectively (p = 0.002). FIB-4 had a positive correlation with baPWV (ρ = 0.29; p < 0.001), mean CIMT (ρ = 0.36; p < 0.001), and maximal CIMT (ρ = 0.31; p < 0.001). Higher FIB-4 remained independently associated with mean CIMT (β = 0.041; p < 0.001), maximal CIMT (β = 0.073; p = 0.001), baPWV (β = 96.4; p = 0.002), and carotid plaque (odds ratio = 1.48; p = 0.002) after controlling for covariates.

Conclusions

In individuals with T2DM, higher FIB-4 was significantly linked to structural and functional indicators of subclinical vascular disease, indicating its possible use as a straightforward risk-enrichment marker for cardiovascular evaluation.

## Introduction

Type 2 diabetes mellitus (T2DM) is a leading chronic metabolic disease, which contributes to significant cardiovascular morbidity and mortality [1,2]. The chronic hyperglycemia, insulin resistance, dyslipidemia, oxidative stress, chronic inflammation, endothelial dysfunction, and advanced glycation seen in people with T2DM lead to increased vascular damage and atherosclerosis [3]. Importantly, subclinical vascular abnormalities may occur before clinically apparent cardiovascular disease, emphasizing the need for simple markers to enhance early cardiovascular risk assessment [4].

There is mounting evidence that hepatic damage and fibrosis are closely linked to metabolic dysfunction in T2DM [5,6]. Hepatic fibrosis can be promoted by insulin resistance, lipid accumulation, oxidative stress, and inflammatory processes, which can also lead to systemic inflammation, endothelial dysfunction, and vascular remodeling [7]. Therefore, hepatic fibrosis can be both a liver defect and a surrogate for systemic cardiometabolic risk [8].

Age, aspartate aminotransferase (AST), alanine aminotransferase (ALT), and platelet count are the components of the simple, non-invasive Fibrosis-4 (FIB-4) index [9,10]. The FIB-4 score helps to estimate the amount of scarring in the liver [9]. Originally used to predict the risk of advanced hepatic fibrosis, FIB-4 has been the focus of increasing studies in relation to cardiovascular disease and vascular abnormalities. It uses parameters that are routinely available in the clinical and laboratory setting, and thus may be useful in risk assessment in resource-limited environments [11]. FIB-4 is an indirect fibrosis risk score, however, and its link with objective measures of subclinical atherosclerosis in T2DM patients has not been fully elucidated yet [12,13]. Brachial-ankle pulse wave velocity (baPWV) is a measure of arterial stiffness, whereas carotid intima-media thickness (CIMT) is a non-invasive indicator of structural alterations in the arteries [14]. Despite the significant prevalence of T2DM and cardiovascular illnesses in South Asian communities, there is little data from these groups, particularly from Pakistan.

Therefore, this study aimed to evaluate the association between the FIB-4 index and subclinical vascular disease in patients with T2DM using mean CIMT as the primary outcome and maximum CIMT, carotid plaque, and baPWV as secondary vascular outcomes. Mean CIMT was selected as the primary measure to reflect overall carotid arterial wall thickness, while maximum CIMT, carotid plaque, and baPWV were assessed as complementary measures of focal carotid changes and arterial stiffness.

## Materials and methods

Study design and setting

This analytical cross-sectional study was conducted at Sheikh Zayed Medical College and Hospital, Rahim Yar Khan, Pakistan, between April 2024 and March 2026 to examine the association between the FIB-4 index and subclinical vascular disease in an adult population with T2DM. Mean CIMT was prespecified as the primary vascular outcome, while maximum CIMT, carotid plaque, and baPWV were evaluated as secondary vascular outcomes. Mean CIMT was selected as the primary measure because it represents overall carotid arterial wall thickness, whereas maximum CIMT and carotid plaque provide complementary information regarding focal carotid arterial changes, and baPWV reflects arterial stiffness.

Study population and sampling

The primary goal of determining the sample size was to evaluate the correlation between the mean CIMT and the FIB-4 index. Based on prior studies of a link between FIB-4 and CIMT in T2DM patients, a cautious correlation coefficient of 0.20 was used to estimate the sample size. The study needed a minimum sample size of 194 individuals with a statistical power of 80% and a two-sided α level of 0.05. The minimum sample size requirement was raised to 214 as it was anticipated that 10% of the data would be missing. To provide for some missing information and increase the accuracy of multivariable estimates, 250 participants were recruited. Participants were recruited using consecutive sampling until the required sample size was reached.

Eligibility criteria

The participants included those with documented T2DM, age ≥18 years, receiving routine diabetes care at Sheikh Zayed Medical College and Hospital, having required clinical and laboratory data to calculate FIB-4, and who gave informed written consent. Patients with type 1 diabetes mellitus, acute hepatitis, advanced chronic liver disease or cirrhosis, active malignancy, severe hematological disorders (platelets), acute infection or systemic inflammation, pregnancy, recent major cardiovascular or cerebrovascular events, and incomplete information for FIB-4 or CIMT calculations were excluded. Patients with excessive alcohol consumption were also excluded to reduce non-metabolic alcohol effects on the FIB-4 index.

Data collection

A standard and pre-tested data collection form was used to collect data. Demographic data, length of diabetes, comorbidities, smoking history, medications, and cardiovascular risk factors were collected via structured interview and medical-record review. Anthropometric measurements used were height, weight, and body mass index (BMI). After an appropriate resting period, blood pressure was obtained using a calibrated or validated device, and the average of repeated measurements were recorded. Systematic collection of potential confounding factors related to both FIB-4 and vascular disease (age, sex, BMI, diabetes duration, HbA1c, blood pressure, lipid profile, renal function, smoking, and lipid-lowering/hypertensive therapy) was performed.

Laboratory assessment

Venous blood samples were obtained following an overnight fast to evaluate AST, ALT, platelet count, fasting blood glucose, HbA1c, total cholesterol, triglycerides, high-density lipoprotein-cholesterol (HDL-C), low-density lipoprotein-cholesterol (LDL-C), serum creatinine, and estimated glomerular filtration rate (eGFR) using established institutional laboratory protocols. Regular internal quality control processes were followed, and laboratory equipment was calibrated adequately.

Fibrosis‐4 calculation

The established FIB-4 index was calculated using age, platelet count, AST, and ALT according to the original published method [9]: $\mathrm{FIB\mathrm{-}4} = \frac{\mathrm{Age\ (years)} \times \mathrm{AST\ (U/L)}} {\mathrm{Platelet\ count\ (10^{9}/L)} \times \sqrt{\mathrm{ALT\ (U/L)}}}$. FIB-4 was analyzed primarily as a continuous variable to preserve the information contained within its full distribution. For secondary categorical analyses, participants were classified as having low (<1.30), intermediate (1.30 to <2.67), or high (≥2.67) FIB-4 values based on previously established thresholds for hepatic fibrosis risk stratification [15]. These cutoffs were originally validated for assessing the probability of advanced hepatic fibrosis, particularly in patients with non-alcoholic fatty liver disease, and were not developed as thresholds for vascular disease [15]. Accordingly, their use in the present study was exploratory and intended to examine whether conventional hepatic fibrosis risk categories were also associated with differences in subclinical vascular measures. Because age is incorporated into the FIB-4 equation and is independently associated with vascular disease, potential collinearity between age and FIB-4 was specifically considered during statistical modeling.

Assessment of carotid intima-media thickness

The primary measure of subclinical atherosclerosis was mean bilateral CIMT. High-resolution B-mode carotid ultrasonography was performed by a trained sonographer using a standardized imaging protocol. Participants were examined in the supine position, and both common carotid arteries were evaluated. CIMT was measured at plaque-free segments of the far wall of each common carotid artery, approximately 1 cm proximal to the carotid bulb. Three measurements were obtained from each side. The mean CIMT for the right and left carotid arteries was calculated separately from these three measurements, and mean bilateral CIMT was calculated as the average of the mean right and mean left CIMT values. Maximum CIMT was recorded separately as a secondary vascular measure representing the greatest focal carotid wall thickness observed during assessment. Carotid plaque was defined as a focal structure protruding into the arterial lumen by at least 0.5 mm, or by ≥50% compared with the surrounding intima-media thickness, or having a thickness >1.5 mm measured from the media-adventitia interface to the intima-lumen interface. To reduce measurement bias, sonographers were blinded to FIB-4 values wherever feasible.

Assessment of brachial-ankle pulse wave velocity

The baPWV was measured as a secondary measurement of arterial stiffness, with the use of a validated automated device and using manufacturer-provided standard protocol. After a proper resting period in the supine position, measurements were obtained. Measurements were averaged if technically acceptable, and the number of participants with technically acceptable baPWV was reported.

Study variables and statistical analysis

The primary outcome was mean bilateral CIMT, and the primary independent variable was FIB-4; the secondary outcomes were maximum CIMT, carotid plaque, and baPWV. Mean CIMT was treated as the primary outcome because it provides an overall measure of carotid arterial wall thickness, whereas maximum CIMT was analyzed as a complementary secondary measure of focal wall thickening. Spearman correlation, multivariable linear regression, and logistic regression were used to evaluate associations; the findings were presented as β/odds ratio (OR), 95% confidence interval (CI), and p-values. Multivariable linear regression was used for the continuous outcomes of mean CIMT, maximum CIMT, and baPWV, while multivariable logistic regression was used for the binary outcome of carotid plaque. Using variance inflation factors and an age-excluded sensitivity analysis, age-related collinearity in the FIB-4 formula was evaluated. The threshold for statistical significance was fixed at p-values <0.05.

Quality control

The recruitment of participants, clinical assessment, laboratory testing, calculation of the FIB-4, and vascular imaging were standardized. Research staff were instructed in data collection procedures, and routine quality control was conducted on the laboratory measurements, while vascular measurements were conducted following a standardized protocol. Before statistical analysis, all data were checked for completeness, consistency, implausible values, and calculation errors.

Ethical considerations

The Sheikh Zayed Medical College and Hospital Institutional Review Board/Ethics Committee granted ethical approval (approval number: 289/IRB/SZMC/SZH), and each participant provided written informed consent. By issuing coded identifying numbers, storing the study data securely, and granting access to only authorized investigators, anonymity was preserved. The Declaration of Helsinki and institutional ethical guidelines were followed when conducting the study.

## Results

Table 1 summarizes the baseline characteristics of the 250 participants. The mean age was 58.7 ± 10.4 years, with 113 (45.2%) aged ≥60 years, and 139 (55.6%) were male. Hypertension was present in 157 (62.8%) participants and dyslipidemia in 168 (67.2%) participants. The mean FIB-4 index was 1.71 ± 0.92. The mean CIMT was 0.78 ± 0.15 mm, and the maximum CIMT was 1.02 ± 0.31 mm. Carotid plaque was present in 71 (28.4%) participants. The mean baPWV was 1,742 ± 348 cm/s.

**Table 1:** Baseline demographic, clinical, laboratory, and vascular characteristics of the study participants. Values are presented as mean ± standard deviation or n (%), as appropriate. BMI = body mass index; BP = blood pressure; HbA1c = glycated hemoglobin; HDL-C = high-density lipoprotein cholesterol; LDL-C = low-density lipoprotein cholesterol; eGFR = estimated glomerular filtration rate; AST = aspartate aminotransferase; ALT = alanine aminotransferase; FIB-4 = Fibrosis-4 index; CIMT = carotid intima-media thickness; baPWV = brachial-ankle pulse wave velocity

Category	Characteristic	Overall (n = 250)
Demographic characteristics	Age, years	58.7 ± 10.4
	Age ≥60 years, n (%)	113 (45.20)
	Male, n (%)	139 (55.60)
	Female, n (%)	111 (44.40)
Clinical characteristics	Duration of diabetes, years	9.1 ± 6.2
	BMI, kg/m²	28.1 ± 4.6
	BMI ≥25 kg/m², n (%)	198 (79.20)
	Current smoking, n (%)	52 (20.80)
	Hypertension, n (%)	157 (62.80)
	Dyslipidemia, n (%)	168 (67.20)
	Systolic BP, mmHg	137.6 ± 18.9
	Diastolic BP, mmHg	82.4 ± 10.7
Glycemic and lipid characteristics	HbA1c, %	8.1 ± 1.7
	Fasting glucose, mg/dL	164.8 ± 52.6
	Total cholesterol, mg/dL	194.6 ± 42.1
	Triglycerides, mg/dL	171.3 ± 86.7
	HDL-C, mg/dL	43.8 ± 10.5
	LDL-C, mg/dL	116.7 ± 34.8
Renal characteristics	Creatinine, mg/dL	1.01 ± 0.31
	eGFR, mL/minute/1.73 m²	78.6 ± 21.7
Hepatic and hematological characteristics	AST, U/L	28.7 ± 11.3
	ALT, U/L	30.9 ± 13.8
	Platelet count, ×10⁹/L	248.6 ± 61.4
FIB-4 index	FIB-4 index	1.71 ± 0.92
Vascular characteristics	Mean CIMT, mm	0.78 ± 0.15
	Maximum CIMT, mm	1.02 ± 0.31
	Carotid plaque, n (%)	71 (28.40)
	baPWV, cm/s	1,742 ± 348

A notable graded rise in vascular measurements across FIB-4 categories is shown in Table 2. The low FIB-4 group included 112 (44.8%) participants, the intermediate group included 119 (47.6%), and the high FIB-4 group included 19 (7.6%). In the low FIB-4 group, the mean CIMT was 0.72 ± 0.11 mm; in the intermediate and high FIB-4 groups, it was 0.81 ± 0.14 mm and 0.91 ± 0.17 mm, respectively (p < 0.001). Similarly, maximum CIMT increased from 0.89 ± 0.24 to 1.07 ± 0.30 and 1.26 ± 0.38 mm (p < 0.001), while carotid plaque was present in 20 (17.9%), 41 (34.5%), and 10 (52.6%) participants, respectively (p = 0.002). baPWV also increased progressively from 1,603 ± 281 to 1,812 ± 327 and 2,041 ± 386 cm/s (p < 0.001).

**Table 2:** Vascular measures according to FIB-4 categories. Values are presented as mean ± standard deviation or n (%), as appropriate. P-values indicate comparisons across the FIB-4 categories. FIB-4 = Fibrosis-4 index; CIMT = carotid intima-media thickness; baPWV = brachial-ankle pulse wave velocity

FIB-4 category	n (%)	FIB-4 index	Mean CIMT, mm	Maximum CIMT, mm	Carotid plaque, n (%)	baPWV, cm/s
Low (<1.30)	112 (44.80)	0.92 ± 0.24	0.72 ± 0.11	0.89 ± 0.24	20 (17.86)	1,603 ± 281
Intermediate (1.30–2.67)	119 (47.60)	1.75 ± 0.36	0.81 ± 0.14	1.07 ± 0.30	41 (34.45)	1,812 ± 327
High (≥2.67)	19 (7.60)	3.21 ± 0.61	0.91 ± 0.17	1.26 ± 0.38	10 (52.63)	2,041 ± 386
Overall	250 (100.00)	1.71 ± 0.92	0.78 ± 0.15	1.02 ± 0.31	71 (28.40)	1,742 ± 348
P-value	—	<0.001	<0.001	<0.001	0.002	<0.001

Table 3 shows significant positive correlations between FIB-4 and all continuous vascular measures. FIB-4 was positively correlated with mean CIMT (Spearman’s ρ = 0.36, p < 0.001), maximum CIMT (ρ = 0.31, p < 0.001), and baPWV (ρ = 0.29, p < 0.001).

**Table 3:** Correlation of the FIB-4 index with measures of subclinical vascular disease. FIB-4 = Fibrosis-4 index; CIMT = carotid intima-media thickness; baPWV = brachial-ankle pulse wave velocity; ρ = Spearman rank correlation coefficient

Vascular measure	Spearman’s (ρ)	P-value
Mean CIMT, mm	0.36	<0.001
Maximum CIMT, mm	0.31	<0.001
baPWV, cm/s	0.29	<0.001

The main multivariable analysis is shown in Table 4. FIB-4 was the primary exposure variable of interest, while age, sex, smoking, diabetes duration, BMI, systolic blood pressure, HbA1c, LDL-C, and eGFR were included as covariates to control for potential confounding. After adjustment for these variables, higher FIB-4 was independently associated with higher mean CIMT (β = 0.041, 95% CI: 0.018-0.064; p < 0.001). The model explained 29% of the variation in mean CIMT (R² = 0.29; adjusted R² = 0.26; overall p < 0.001). Assessment of multicollinearity showed variance inflation factor (VIF) values ranging from 1.07 to 2.34. The highest VIF values were observed for age (2.34) and FIB-4 (2.18), while all remaining predictors had VIF values below 1.50. As all VIF values were below 5, no problematic multicollinearity was identified in the fully adjusted model.

**Table 4:** Independent association of FIB-4 index with mean CIMT in patients with T2DM. FIB-4 was the primary exposure variable; the remaining variables were included as covariates for multivariable adjustment. T2DM = type 2 diabetes mellitus; FIB-4 = Fibrosis-4 index; CIMT = carotid intima-media thickness; SE = standard error; CI = confidence interval; LDL-C = low-density lipoprotein cholesterol; HbA1c = glycated hemoglobin; BMI = body mass index; BP = blood pressure; eGFR = estimated glomerular filtration rate; β = regression coefficient; R² = coefficient of determination; VIF = variance inflation factor

Variable	β coefficient	SE	95% CI	P-value	VIF
FIB-4 index	0.041	0.012	0.018–0.064	<0.001	2.18
Age, years	0.002	0.001	0.001–0.003	0.006	2.34
Male sex	0.018	0.012	−0.006–0.042	0.141	1.07
LDL-C, mg/dL	0.000	0.000	−0.000–0.001	0.084	1.16
HbA1c, %	0.008	0.005	−0.002–0.018	0.118	1.12
Current smoking	0.021	0.013	−0.005–0.047	0.109	1.09
BMI, kg/m²	0.003	0.002	−0.001–0.007	0.163	1.24
Systolic BP, mmHg	0.001	0.000	0.000–0.002	0.021	1.31
eGFR, mL/minute/1.73 m²	−0.001	0.000	−0.002–0.000	0.048	1.38
Diabetes duration, years	0.002	0.001	0.000–0.004	0.052	1.42

Table 5 shows the adjusted associations between FIB-4 and secondary vascular outcomes. After adjustment for age, sex, BMI, diabetes duration, HbA1c, systolic blood pressure, LDL-C, current smoking, and eGFR, higher FIB-4 was independently associated with greater maximum CIMT (β = 0.073, 95% CI: 0.031-0.115; p = 0.001) and higher baPWV (β = 96.4, 95% CI: 34.7-158.1; p = 0.002). Higher FIB-4 was also associated with increased odds of carotid plaque (OR = 1.48, 95% CI: 1.16-1.89; p = 0.002).

**Table 5:** Multivariable associations between FIB-4 and secondary vascular outcomes. Maximum CIMT and baPWV were analyzed using multivariable linear regression, while carotid plaque was analyzed using multivariable logistic regression. All models were adjusted for age, sex, BMI, diabetes duration, HbA1c, systolic blood pressure, LDL-C, current smoking, and eGFR. FIB-4 = Fibrosis-4 index; CIMT = carotid intima-media thickness; baPWV = brachial-ankle pulse wave velocity; β = regression coefficient; CI = confidence interval; OR = odds ratio; BMI = body mass index; HbA1c = glycated hemoglobin; LDL-C = low-density lipoprotein cholesterol; eGFR = estimated glomerular filtration rate

Outcome	Effect measure	Estimate	95% CI	P-value
Maximum CIMT	β coefficient	0.073	0.031–0.115	0.001
baPWV	β coefficient	96.4	34.7–158.1	0.002
Carotid plaque	Odds ratio	1.48	1.16–1.89	0.002

Table 6 presents the sensitivity analysis evaluating the potential influence of age on the association between FIB-4 and mean CIMT. The association remained significant in the unadjusted model (β = 0.059, 95% CI: 0.039-0.079; p < 0.001); in the multivariable model adjusted for sex, BMI, diabetes duration, HbA1c, systolic blood pressure, LDL-C, smoking, and eGFR but excluding age (β = 0.050, 95% CI: 0.029-0.071; p < 0.001); and in the fully adjusted model that additionally included age (β = 0.041, 95% CI: 0.018-0.064; p < 0.001). The persistence of the association across these models indicates that the observed FIB-4-CIMT association was not explained solely by the age component of the FIB-4 calculation.

**Table 6:** Sensitivity analysis of the association between FIB-4 and mean CIMT. FIB-4 = Fibrosis-4 index; CIMT = carotid intima-media thickness; CI = confidence interval; BMI = body mass index; HbA1c = glycated hemoglobin; BP = blood pressure; LDL-C = low-density lipoprotein cholesterol; eGFR = estimated glomerular filtration rate; β = regression coefficient; R² = coefficient of determination

Model	Adjustment	β coefficient for FIB-4	95% CI	P-value	R²
Model 1	Unadjusted	0.059	0.039–0.079	<0.001	0.13
Model 2	Adjusted for Gender, BMI, diabetes duration, HbA1c, systolic BP, LDL-C, smoking, and eGFR; age excluded	0.050	0.029–0.071	<0.001	0.27
Model 3	Fully adjusted model including age	0.041	0.018–0.064	<0.001	0.29

## Discussion

The present study demonstrated a positive association between the FIB-4 index and subclinical vascular disease among 250 patients with T2DM. Vascular measures showed a graded pattern across FIB-4 categories, with mean CIMT increasing progressively from the low to the high FIB-4 group (p < 0.001). Carotid plaque was present in 20 (17.9%), 41 (34.5%), and 10 (52.6%) participants in the low, intermediate, and high FIB-4 groups, respectively (p = 0.002). This graded pattern supports the possibility that a higher fibrosis risk score identifies a cardiometabolic phenotype characterized by greater subclinical vascular burden. A recent meta-analysis of 2,658 patients with T2DM similarly reported increasing carotid atherosclerosis across FIB-4 categories, with higher CIMT and carotid plaque remaining associated with FIB-4 ≥2.67 after adjustment [16].

Correlation analysis further supported these findings, with FIB-4 showing positive associations with baPWV, maximum CIMT, and mean CIMT. These findings are consistent with Watanabe et al. (2022), who studied 253 patients with T2DM and reported a significant association between FIB-4 and CIMT (r = 0.241, p < 0.001). They also demonstrated associations between FIB-4 and aortic arch and carotid artery calcification, suggesting that a higher FIB-4 may reflect vascular injury beyond hepatic abnormalities [17]. The somewhat stronger association with mean CIMT in the present study may reflect differences in clinical setting, participant characteristics, or vascular assessment methods.

The categorical findings also demonstrated a dose-response pattern, with carotid plaque occurring more frequently in participants with intermediate and high FIB-4 values than in those with low values. Maximum CIMT and baPWV similarly increased across the three categories. These findings are comparable with those of Miao et al. (2025), who reported greater carotid atherosclerosis, CIMT, and carotid plaque among patients with higher FIB-4 values. However, the discriminatory performance of FIB-4 was modest (area under the curve (AUC) 0.561 for increased CIMT and 0.580 for carotid plaque), indicating that FIB-4 should be considered a risk-enrichment marker rather than a diagnostic tool [16].

The observed associations persisted after adjustment for relevant vascular and metabolic factors. Each unit increase in FIB-4 was independently associated with higher mean CIMT, maximum CIMT, baPWV, and the odds of carotid plaque. Watanabe et al. similarly reported an independent relationship between FIB-4 and CIMT (β = 0.241, p = 0.004), together with other markers of arterial damage [17]. Collectively, these findings suggest that the relationship between FIB-4 and subclinical vascular abnormalities may not be fully explained by conventional glycemic, lipid, blood pressure, smoking, or renal factors.

These findings are also consistent with broader cardiovascular evidence. A systematic review and meta-analysis of 25,252 patients found that higher FIB-4 was associated with cardiovascular events (hazard ratio (HR) = 1.75, 95% CI: 1.53-2.00) [18], while another analysis reported a 15% increase in cardiovascular event risk for each unit increase in FIB-4 (HR = 1.15, 95% CI: 1.06-1.24). Bang et al. (2024) additionally reported an association between higher FIB-4 and greater estimated atherosclerotic cardiovascular disease (ASCVD) risk, with 44.5% of participants in the high/very high FIB-4 categories having an estimated ASCVD risk ≥20% [19]. The present findings extend this literature by demonstrating associations between FIB-4 and directly measured structural and functional vascular phenotypes in patients with T2DM, rather than relying solely on predicted cardiovascular risk or clinical cardiovascular outcomes.

Although age is incorporated into the FIB-4 equation, a sensitivity analysis was performed to assess the robustness of the primary association and the potential influence of age. The association between FIB-4 and mean CIMT remained significant in the unadjusted model (β = 0.059, 95% CI: 0.039-0.079; p < 0.001); in the multivariable model adjusted for sex, BMI, diabetes duration, HbA1c, systolic blood pressure, LDL-C, smoking, and eGFR but excluding age (β = 0.050, 95% CI: 0.029-0.071; p < 0.001); and in the fully adjusted model that additionally included age (β = 0.041, 95% CI: 0.018-0.064; p < 0.001). The persistence of the association across these models suggests that the relationship between FIB-4 and CIMT was not explained solely by the age component of FIB-4. However, because this was a cross-sectional, hospital-based study, causal inferences cannot be made, and FIB-4 should not be interpreted as a direct measure of atherosclerotic plaque or as a substitute for dedicated assessment of hepatic fibrosis. Nevertheless, these findings support the potential role of FIB-4 as a low-cost risk-enrichment marker for identifying patients with T2DM who may benefit from more intensive cardiovascular risk assessment [16,20].

Strengths and limitations

One of the strengths of this study is the evaluation of the easily accessible and low-cost FIB-4 index alongside objective measures of subclinical vascular disease, including CIMT, carotid plaque, and baPWV, in a well-defined sample of 250 patients with T2DM. Because FIB-4 is calculated from routinely available clinical and laboratory parameters, it may be particularly useful for risk enrichment in resource-limited settings, although its clinical utility for this purpose requires further validation. The findings were further strengthened by standardized vascular assessments, multivariable adjustment for major cardiometabolic confounders, and a sensitivity analysis addressing the potential influence of age incorporated into the FIB-4 formula.

Several limitations should also be acknowledged. The cross-sectional design precludes determination of temporal relationships or causal inference, while the single-center setting and relatively modest sample size may limit the generalizability of the findings. In particular, the high FIB-4 category included only 19 participants, which may reduce the precision and stability of estimates in this subgroup. Furthermore, FIB-4 is an indirect fibrosis risk score rather than a direct measurement of hepatic fibrosis, and residual confounding from unmeasured factors cannot be excluded. Larger prospective, multicenter studies incorporating validated measures of hepatic fibrosis are needed to confirm these findings and evaluate their prognostic significance.

## Conclusions

This hospital-based cohort of patients with T2DM showed that higher FIB-4 was independently and significantly associated with higher mean and maximum CIMT, higher carotid plaque prevalence, and higher baPWV, suggesting a uniform link between fibrosis risk status and structural and functional vascular abnormalities. These associations persisted after adjusting for major cardiometabolic risk factors and were also observed in sensitivity analyses to control for the possible confounding effects of age. The incremental steps in vascular measures according to the FIB-4 categories further support a graded association between FIB-4 and subclinical vascular disease. FIB-4 should not be used as a direct measure of hepatic fibrosis or atherosclerosis. However, because it is inexpensive, easily calculated, and based on routinely available clinical and laboratory data, it may have potential as a risk-enrichment marker for identifying patients with T2DM who warrant further cardiovascular risk evaluation. Given the cross-sectional design of the present study, these findings do not establish that FIB-4-guided cardiovascular assessment or intervention improves clinical outcomes. Further prospective, multicenter studies are needed to validate these associations and determine whether incorporation of FIB-4 into cardiovascular risk assessment provides incremental prognostic or clinical benefit.
